# Continuous regional arterial infusion versus intravenous administration of the protease inhibitor nafamostat mesilate for predicted severe acute pancreatitis: a multicenter, randomized, open-label, phase 2 trial

**DOI:** 10.1007/s00535-019-01644-z

**Published:** 2019-11-22

**Authors:** Morihisa Hirota, Tooru Shimosegawa, Katsuya Kitamura, Kazunori Takeda, Yoshifumi Takeyama, Toshihiko Mayumi, Tetsuhide Ito, Mamoru Takenaka, Eisuke Iwasaki, Hirotaka Sawano, Etsuji Ishida, Shin Miura, Atsushi Masamune, Yousuke Nakai, Akira Mitoro, Hiroyuki Maguchi, Kenji Kimura, Tsuyoshi Sanuki, Tetsuya Ito, Hiroki Haradome, Kazuto Kozaka, Toshifumi Gabata, Keisho Kataoka, Masahiko Hirota, Shuji Isaji, Ryoji Nakamura, Koki Yamagiwa, Chie Kayaba, Koji Ikeda

**Affiliations:** 1 Division of Gastroenterology and Hepatology, Tohoku Medical and Pharmaceutical University, 1-15-1, Fukumuro, Miyagino-ku, Sendai, Miyagi 9838536 Japan; 2 Department of Gastroenterology, South Miyagi Medical Center, 28-1 Nishi, Ohgawara, Miyagi 9891253 Japan; 3 Division of Gastroenterology, Department of Medicine, Showa University School of Medicine, 1-5-8, Hatanodai, Shinagawa-ku, Tokyo, 1428666 Japan; 4 Present Address: Department of Gastroenterology and Hepatology, Tokyo Medical University Hachioji Medical Center, 1163, Tatemachi, Hachioji-Shi, Tokyo 1930998 Japan; 5 Miyagi Branch, Health Insurance Claims Review & Reimbursement Services, 5-1-27, Tsutsujigaoka, Miyagino-ku, Sendai, Miyagi 9838504 Japan; 6 Department of Surgery, Kindai University, Faculty of Medicine, 377-2, Ohno-Higashi, Osaka-Sayama, Osaka 5898511 Japan; 7 Department of Emergency Medicine, University of Occupational and Environmental Health, 1-1, Iseigaoka, Yahatanishi-ku, Kitakyushu, Fukuoka 8078555 Japan; 8 Department of Gastroenterology and Hepatology, International University of Health and Welfare Graduate School of Medicine, Neuroendocrine Tumor Center, Fukuoka Sanno Hospital, 3-6-45, Momochihama, Sawara-ku, Fukuoka, 8140001 Japan; 9 Department of Gastroenterology and Hepatology, Kindai University, Faculty of Medicine, 377-2, Ohno-Higashi, Osaka-Sayama, Osaka 5898511 Japan; 10 Division of Gastroenterology and Hepatology, Department of Internal Medicine, Keio University School of Medicine, 35, Shinanomachi, Shinjuku-ku, Tokyo, 1608582 Japan; 11 Senri Critical Care Medical Center, Osaka Saiseikai Senri Hospital, 1-1-6, Tsukumodai, Suita, Osaka 5650862 Japan; 12 Department of Gastroenterology, Kurashiki Central Hospital, 1-1-1, Miwa, Kurashiki, Okayama 7108602 Japan; 13 Division of Gastroenterology, Tohoku University Graduate School of Medicine, 1-1, Seiryo, Aoba-ku, Sendai, Miyagi 9808574 Japan; 14 Department of Gastroenterology, Graduate School of Medicine, The University of Tokyo, Tokyo, Japan; 15 Department of Endoscopy and Endoscopic Surgery, Graduate School of Medicine, The University of Tokyo, 7-3-1, Hongo, Bunkyo-ku, Tokyo, 1138655 Japan; 16 Third Department of Internal Medicine, Nara Medical University, 840, Shijo-cho, Kashihara, Nara 6348522 Japan; 17 Center for Gastroenterology, Teine-keijinkai Hospital, 1-12-1-40, Maeda, Teine-ku, Sapporo, 0068555 Japan; 18 Department of Gastroenterology, National Hospital Organization Sendai Medical Center, 2-11-12, Miyagino, Miyagino-ku, Sendai, Miyagi 9838520 Japan; 19 Department of Gastroenterology, Kita-Harima Medical Center, 926-250, Ichiba-cho, Ono, Hyogo 6751392 Japan; 20 Department of Internal Medicine, Gastroenterology, Shinshu University Hospital, 3-1-1, Akashi, Matsumoto, Nagano 3908621 Japan; 21 Present Address: Division of Gastroenterology, Nagano Red Cross Hospital, 5-22-1, Wakasato, Nagano, 3808582 Japan; 22 Department of Radiological Advanced Medicine, Kitasato University School of Medicine, 1-15-1, Kitasato, Minami-ku, Sagamihara, Kanagawa 2520375 Japan; 23 Department of Radiology, Kanazawa University, Graduate School of Medical Sciences, 13-1, Takaramachi, Kanazawa, Ishikawa 9208641 Japan; 24 Department of Gastroenterology, Otsu Municipal Hospital, 2-9-9, Motomiya, Otsu, Shiga 5200804 Japan; 25 Department of Surgery, Kumamoto Regional Medical Center, 5-16-10, Honjou, Chuou-ku, Kumamoto, 8600811 Japan; 26 Department of Hepatobiliary Pancreatic and Transplant Surgery, Mie University Graduate School of Medicine, 2-174, Edobashi, Tsu, Mie 5148507 Japan; 27 Inter Scientific Research Co., Ltd, 3-14-1, Higashinakano, Nakano-ku, Tokyo, 1640003 Japan; 28 Department of Development Promotion, Clinical Research, Innovation, Education Center, Tohoku University Hospital, 1-1, Seiryo, Aoba-ku, Sendai, Miyagi 9808574 Japan

**Keywords:** Acute pancreatitis, Protease inhibitor, Continuous regional arterial infusion, Pancreatic necrosis, Analgesic

## Abstract

**Background:**

Continuous regional arterial infusion (CRAI) of protease inhibitor nafamostat mesilate (NM) is used in the context of predicted severe acute pancreatitis (SAP) to prevent the development of pancreatic necrosis. Although this therapy is well known in Japan, its efficacy and safety remain unclear.

**Methods:**

This investigator-initiated and -driven, multicenter, open-label, randomized, controlled trial (UMIN000020868) enrolled 39 patients with predicted SAP and low enhancement of the pancreatic parenchyma on computed tomography (CT). Twenty patients were assigned to the CRAI group, while 19 served as controls and were administered NM at the same dose intravenously (IV group). The primary endpoint was the development of pancreatic necrosis as determined by CT on Day 14, judged by blinded central review.

**Results:**

There was no difference between the CRAI and IV groups regarding the percentages of participants who developed pancreatic necrosis (more than 1/3 of the pancreas: 25.0%, range 8.7–49.1% vs. 15.8%, range 3.4–39.6%, respectively, *P* = 0.694; more than 2/3 of the pancreas: 20%, range 5.7–43.7% vs. 5.3%, range 0.1–26.0%, respectively, *P* = 0.341). The early analgesic effect was evaluated based on 24-h cumulative fentanyl consumption and additional administration by intravenous patient-controlled analgesia. The results showed that the CRAI group used significantly less analgesic. There were two adverse events related to CRAI, namely bleeding and splenic infarction.

**Conclusions:**

CRAI with NM did not inhibit the development of pancreatic necrosis although early analgesic effect of CRAI was superior to that of IV. Less-invasive IV therapy can be considered a viable alternative to CRAI therapy.

**Electronic supplementary material:**

The online version of this article (10.1007/s00535-019-01644-z) contains supplementary material, which is available to authorized users.

## Introduction

Acute pancreatitis (AP) is an abdominal inflammatory disorder. A key event in its initiation and progression is the ectopic activation of the protease trypsin in the acinar cells of the pancreas [1–3]. Trypsin triggers the sequential activation of other pancreatic digestive enzymes, leading to autodigestion. The injured acinar cells release trypsin and damage-associated molecular pattern molecules (DAMPs), which cause pancreatic and peripancreatic necrosis as well as organ failure, mainly as a result of vascular injury [3, 4]. Although the severity of AP is defined by the presence of persistent organ failure [5], other reports have suggested that the extent of pancreatic necrosis is closely related to the morbidity and mortality of severe AP (SAP) [6–8].

Nafamostat mesilate (NM) and gabexate mesilate (GM) are synthetic protease inhibitors that were developed to improve drug distribution into the pancreas relative to the previously used aprotinin. Both drugs effectively inhibit trypsin. Moreover, as long as their concentrations are maintained at a sufficiently high level, their anticoagulant effect has been expected to prevent pancreatic necrosis by restoring pancreatic perfusion through the dissolution of thrombus in regional vessels injured by trypsin and DAMPs [3, 9]. However, it has been thought that the local circulatory collapse caused by AP might make it difficult to maintain high concentrations in the pancreatic tissue following intravenous administration [9]. In 1996, Takeda et al. first reported the therapeutic efficacy of continuous regional arterial infusion (CRAI) of NM in patients with SAP; this was a novel drug delivery system to supply NM directly to the pancreas [10]. Since then, CRAI therapy has become widespread and is now practiced throughout Japan. Animal models showed that following CRAI of a proteinase inhibitor, the drug concentration in the pancreatic tissue was 5–32 times higher than that in animals treated with intravenous administration; further, CRAI reduced mortality and improved pancreatic histopathological outcomes [11–13]. Thus far, there has been only one prospective, randomized, controlled study of CRAI administration of NM, reported from Poland. Piaścik et al. demonstrated that several outcomes, including C-reactive protein (CRP) levels, computed tomography severity index (CTSI) scores, the use of additional antibacterial agents and urgent surgical interventions, and mortality, were significantly improved in patients treated with CRAI of NM relative to non-CRAI controls [14]. Their study group was treated with CRAI of NM as well as with the antibiotic imipenem, while controls were treated only with intravenous imipenem. In other words, NM was not administered to controls. Therefore, their study did not address whether invasive CRAI treatment was actually superior to the less-invasive intravenous administration of NM with regard to clinical efficacy and safety in patients with predicted SAP. Recently, a few large-scale retrospective studies were reported. Hamada et al. demonstrated that CRAI therapy with a protease inhibitor was not effective in reducing in-hospital mortality rate [15]. Horibe et al. showed that CRAI was not associated with reductions in mortality, infection rate, or need for surgical intervention [16]. On the other hand, Endo et al. found that CRAI was significantly associated with reduced in-hospital mortality in patients with SAP [17]. The efficacy of CRAI therapy with a protease inhibitor is therefore controversial.

The current open-label, randomized, controlled phase 2 trial was designed to demonstrate the clinical efficacy and safety of CRAI therapy with NM in patients with predicted SAP using a control group treated with continuous IV administration of NM at the same dose as the study group. The primary endpoint was the percentage of participants in each group with a large area of pancreatic necrosis after the study treatment, as determined by blinded central review. The secondary endpoints were outcomes related to inflammation, pain, morbidity, and mortality.

## Methods

### Trial design and oversight

An investigator-initiated and -driven, multicenter, open-label, randomized, controlled phase 2 superiority trial was performed in seven university hospitals and five high-level medical centers in Japan. We prepared the protocol and conducted the trial under the guidance of the Pharmaceutical and Medical Devices Agency (PMDA), and performed the research in accordance with the principles of the Declaration of Helsinki and good clinical practice (GCP) guidelines. The protocol was approved by the institutional review board of Tohoku University Hospital and by those of the participating medical institutions. The registration number of the trial is UMIN000020868.

To demonstrate the clinical usefulness of CRAI of NM in patients with predicted SAP, study participants were randomly assigned in a 1:1 ratio either to a group treated by CRAI of NM (CRAI group) or to a group treated by IV NM (IV group). The study drug, NM, was administered to both groups at 240 mg/day for 5 days. Clinical efficacy and safety were compared between the two groups. Contrast-enhanced CT was performed 14 days after the start of the study drug administration (Day 14). Day 1 was defined as the first 24 h after the start of the study drug administration. The primary endpoint of the trial was defined as the percentage of patients who developed a large extent of pancreatic necrosis (as defined below). Three diagnostic imaging specialists in pancreatic disease who were blinded to the clinical and allocation information evaluated the CT images. Auditing, monitoring, data management, and support for imaging analysis were commissioned to Micron Corporation.

### Study participants

AP was diagnosed if at least two of the following three clinical features were present: typical abdominal pain, abnormally high levels of serum pancreatic enzymes, and characteristic findings of AP on cross-sectional abdominal imaging.

**Fig. 1:**
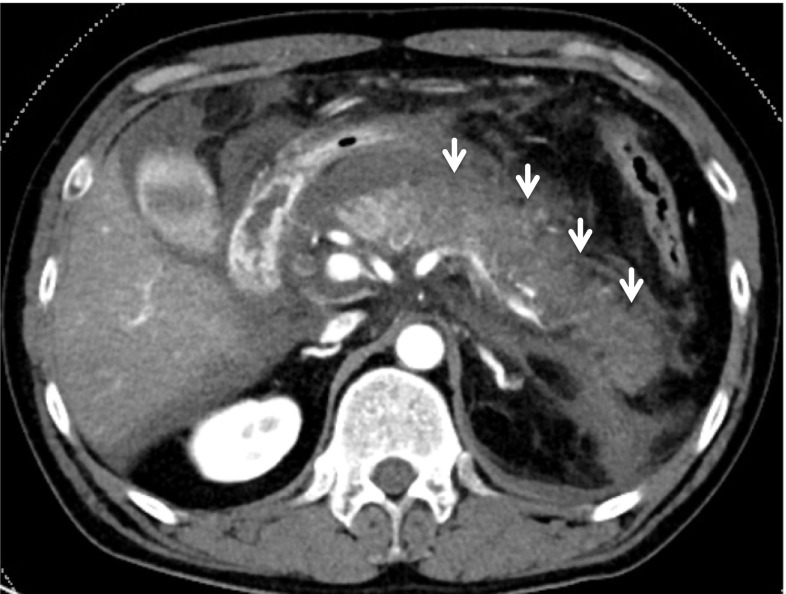
A contrast-enhanced CT image obtained within 48 h after the onset of AP. A CT image of a 46-year-old man shows LEPP (arrows) in the pancreatic body and tail with acute peripancreatic fluid collection. Imaging findings of this patient satisfied the inclusion criteria of this trial

Patients were eligible for this trial if they were diagnosed with SAP according to the Japanese severity criteria: low enhancement of the pancreatic parenchyma (LEPP) in at least one of three pancreatic sections on contrast-enhanced CT (grade 2 or 3) within 48 h after the onset of abdominal pain (Fig. 1) [18]. LEPP was defined if the mean CT value of a maximum region of interest (ROI) in one of three pancreatic sections was less than 70 Hounsfield units (HU). CT images were collected later and inspected by blinded central review. Study drug administration was required to begin within 24 h after the CT.

Patients were not enrolled in this trial if they met at least one of following criteria: treatment with CRAI of a protease inhibitor or anticoagulant agent before informed consent was obtained; IV administration of NM at 240 mg/day or more before informed consent was obtained; inability to specify the onset of abdominal pain; suspected malignancy of the pancreas; history of pancreatectomy; estimated glomerular filtration rate of less than 30 mL/min/1.73 m^2^; serum potassium level of 5.5 mEq/L or higher; contrast agent allergy; pregnancy; age less than 20, or 80 and over; and deemed unsuitable for study participation by an investigator due to serious comorbidity.

In principle, written informed consent was obtained from both the patient and an acceptable legal representative, but if the patient him- or herself had difficulty in communication, the consent was obtained from the legal representative only.

### Trial procedures

Patients with AP who satisfied the selection criteria and did not meet any exclusion criteria were enrolled in this trial after providing written informed consent. They were randomly assigned in a 1:1 ratio either to the CRAI group or the IV group. Randomization was performed by dynamic allocation (minimization method) with the following allocation factors: study facility, etiology (alcoholic), and contrast-enhanced CT grade 3. The study drug NM was purchased from TORII Pharmaceutical. A single catheter was inserted into the inguinal artery of a patient assigned to the CRAI group. The tip was placed in a suitable position in an artery that effectively perfused a region of the pancreas with low enhancement, for instance the celiac artery. In patients assigned to the IV group, the study drug was injected into a peripheral or central vein. The study drug was dissolved in 5% glucose. The antibacterial drug meropenem was intravenously administered to all participants as a concomitant drug at a dose of 1.5 g/day. Pain was managed by intravenous injection of fentanyl by patient-controlled analgesia (IV-PCA) using a previously established protocol (Suppl.). Patients received IV-PCA if their pain was rated 4 or higher by a numerical rating scale (NRS), and their state of consciousness was estimated to be between − 1 and + 1 by the Richmond Agitation-Sedation Scale (RASS). General treatments for AP, for instance, fluid management, enteral nutrition, and breathing and circulation management, were carried out in accordance with existing clinical practice guidelines [19, 20]. Contrast-enhanced CT was performed 2 weeks after the start of NM administration to determine the extent of pancreatic necrosis. All the CT images were collected and analyzed by three radiologists who were blinded to the clinical and allocation information.

### Endpoints

The primary endpoint was a comparison of the percentage of participants in the CRAI and IV groups who developed a large extent of pancreatic necrosis, variably defined in two different analyses as more than one-third of the pancreas or more than two-thirds of the pancreas, as shown by contrast-enhanced CT on Day 14. The imputation of missing data for the primary endpoint was defined in the protocol in advance for participants who could not undergo contrast-enhanced CT on Day 14 due to progressive and persistent renal failure or death.

The following were adopted as secondary endpoints: CTSI estimated by CT images on Day 14; a comparison of 24-hour cumulative fentanyl consumption (CFC) and additional fentanyl administration (AFA) on Day 2, Day 3, and Day 4 in participants who received IV-PCA; the percentage of participants diagnosed with SAP according to the modified Marshall score defined in the revised Atlanta classification [5] between Day 1 and Day 5; the highest prognostic score according to the Japanese severity classification between Day 1 and Day 5; the highest level of CRP between Day 1 and Day 5; the duration of SIRS positivity between Day 1 and Day 5; necrosectomy rate; and mortality up to Day 90.

### Patient safety

For safety reasons, the clinical laboratory data of all participants were evaluated once a day and vital signs were measured at least three times a day for the entire duration of the study drug administration. Reports of adverse events were collected until Day 14. In participants assigned to the CRAI group, catheter-related complications, specifically bleeding from the insertion site, damage to the catheter, severe subcutaneous hematoma, and the circulatory state of the catheterized leg were checked once a day during CRAI therapy. The CRAI treatment was stopped if any of the following criteria were met: occurrence of a serious adverse event, occurrence of serious catheter-related injuries, withdrawal of consent by the participant or the legal acceptable representative, discovery after enrollment that the participant failed to meet eligibility criteria, and participant death. In the case of extremely serious adverse events related to the study drug or other study treatment, the data and safety evaluation committee would consider aborting the trial.

### Statistical analysis

This study was planned as a phase 2 trial for the purpose of collecting data for future verification. Therefore, the sample size was the maximum number of participants who could be enrolled during the implementation period of about 1 year. SAP with early-phase pancreatic ischemia involving more than 30% of the pancreas is relatively rare and accounts for less than 10% of AP cases [21]. Moreover, the number of patients who could satisfy the entry criteria and provide informed consent was expected to be small. The sample size of this trial was defined 20 for each group.

All data analyses were performed in accordance with a pre-established protocol. The full analysis set (FAS) was the target population for evaluating the effectiveness of this trial. The primary endpoint was the percentage of patients with a large extent of pancreatic necrosis; the 95% confidence interval (CI) was calculated for each treatment group, and differences between groups were tested by Fisher’s exact test. The secondary endpoints were analyzed as follows. For CTSI, 24-h CFC and AFA, highest prognostic score, and highest CRP level, the 95% CI of summary statistics and the mean value for each assignment group were calculated; also, the differences in mean values between the two groups were tested by Student’s *t* test. The percentages of severe cases determined by the modified Marshall score were determined for the two groups, along with the 95% CIs, and the differences between the groups were tested by Fisher’s exact test. For the duration of SIRS positivity, the medians and 95% CIs were calculated, and the differences between the groups were tested by the Mann–Whitney *U* test. For necrosectomy, the ratio of the number of patients in each group who underwent necrosectomy and the 95% CIs of these ratios were calculated, and the differences between the groups were tested by Fisher’s exact test. For overall survival from the start of the study drug administration to Day 90, the survival rates were calculated for each assignment group using the Kaplan–Meier method on Day 30, Day 60, and Day 90, and comparison between the groups was performed with the log-rank test. A two-sided P value of less than 0.05 was considered to indicate statistical significance. Computations were performed with the use of SAS software (version 8.4).

### Role of the funding source

The sponsor of the trial had no role in study design, data collection, data analysis, interpretation of the results, or writing of the manuscript. This trial was funded by a Japan Agency for Medical Research and Development (AMED) subsidy. IV-PCA apparatuses were lent free of charge during the study period from Smiths Medical Japan.

## Results

### Enrollment and randomization

**Fig. 2:**
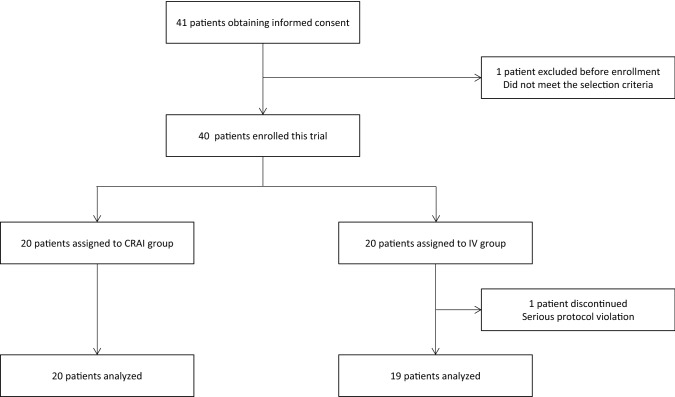
Case diagram

From June 2016 to December 2017, consent was obtained from 41 patients who met the trial selection criteria. One patient was excluded from the trial before enrollment because the study drug administration could not start within 24 h after contrast-enhanced CT. Of the 40 patients who were enrolled, 39 completed the trial. One participant in the IV group was withdrawn from the trial on Day 14 due to a serious protocol violation, and was excluded from all analyses (Fig. 2). The CRAI and IV groups did not show significant differences for all baseline characteristics (Table 1).

**Table 1:** Characteristics of the participants at baseline

	CRAI (*n* = 20)	IV (*n* = 19)	*P* value
Male sex	85.0% (17)	84.2% (16)	1.000
Age (years)	52.0 ± 17.7	58.1 ± 14.0	0.245
Body mass index, kg/m^2^	23.7 ± 5.1	22.9 ± 2.4	0.553
Cause of pancreatitis			
Alcohol	55.0% (11)	47.4% (9)	
Gallstones	20.0% (4)	21.1% (4)	
Other	25.0% (5)	31.5% (6)	
Contrast-enhanced CT grade			0.623
Grade 2	55.0% (11)	63.2% (12)	
Grade 3	45.0% (9)	36.8% (7)	
Prognostic score	1.9 ± 1.6	1.3 ± 1.3	0.222
APACHE II score	8.6 ± 4.1	8.3 ± 3.7	0.797
Modified Marshall score			0.962
4	0.0% (0)	0.0% (0)	
3	5.0% (1)	5.3% (1)	
2	10.0% (2)	10.5% (2)	
0–1	80.0% (16)	78.9% (15)	
No data	5.0% (1)	5.3% (1)	
SIRS			
Temperature > 38 °C	15.0% (3)	5.3% (1)	0.337
Respiration rate > 20/min	70.0% (14)	52.6% (10)	0.313
Pulse > 90 beats per min	60.0% (12)	31.6% (6)	0.103
WBC > 12,000/µL or < 3000/µL	45.0% (9)	47.4% (9)	1.000
CRP (mg/dL)	15.2 ± 11.6	9.8 ± 9.7	0.137
NRS of all participants	4.4 ± 3.3	4.5 ± 3.2	0.871
NRS of participants receiving IV-PCA (N)	5.6 ± 2.9 (9)	5.7 ± 2.7 (11)	0.897
Time from onset of AP to drug administration (h)	37.3 ± 13.7	32.9 ± 16.1	0.368

Data are shown as percentage (*n*) or mean ± standard deviation. Contrast-enhanced CT was performed within 48 h after onset of AP. APACHE II is the Acute Physiology and Chronic Health Evaluation score. NRS is Numerical Rating Scale, a simple scale in which participants rated their pain from 0 (no pain) to 10 (worst pain). The modified Marshall score [5] shows the score for the respiratory system because no participants scored 2 or more for the renal and cardiovascular systems before enrollment

### Outcomes

#### Primary endpoint

Thirty-eight participants underwent contrast-enhanced CT on Day 14. One participant in the CRAI group could not undergo contrast-enhanced CT on Day 14 due to renal failure, and was judged to be positive for the primary endpoint by the previously defined requirement for imputation of missing data. Consequently, eight participants were judged to have developed pancreatic necrosis involving more than one-third of the pancreas; this occurred in 5 of 20 participants in the CRAI group and 3 of 19 participants in the IV group. No superiority of CRAI was demonstrated. Moreover, five participants were judged to have developed pancreatic necrosis involving more than two-thirds of the pancreas; this occurred in 4 of 20 participants in the CRAI group and 1 of 19 participants in the IV group. Again, no superiority of CRAI group was demonstrated (Table 2).

**Table 2:** Primary and secondary endpoints

	CRAI (*n* = 20)	IV (*n* = 19)	Difference	*P*
Primary endpoint				
Pancreatic necrosis involving more than 1/3 of the pancreas	25.0% (5), 8.7–49.1%	15.8% (3), 3.4–39.6%	− 9.2%, − 39.9 to 20.9%	0.694
Pancreatic necrosis involving more than 2/3 of the pancreas	20.0% (4), 5.7–43.7%	5.3% (1), 0.1–26.0%	− 14.7%, − 44.5 to 15.9%	0.341
Secondary endpoints				
CTSI	5.3 ± 2.8, 4.0–6.7	5.2 ± 1.8, 4.3–6.0	0.2, − 1.4 to 1.7	0.837
24-h CFC				
Day 2 (N)	0.46 ± 0.21 (9), 0.30–0.62	0.85 ± 0.42 (11), 0.56–1.13	− 0.39, − 0.72 to − 0.06	0.021
Day 3 (N)	0.50 ± 0.12 (6), 0.37–0.62	0.95 ± 0.58 (10), 0.53–1.36	− 0.45, − 0.87 to − 0.02	0.040
Day 4 (N)	0.45 ± 0.16 (6), 0.28–0.62	0.81 ± 0.69 (9), 0.28–1.34	− 0.3613, − 0.90 to 0.18	0.163
24-h AFA				
Day 2 (N)	0.7 ± 1.0 (9), − 0.1 to 1.4	4.0 ± 3.9 (11), 1.4–6.6	− 3.3, − 6.0 to − 0.7	0.018
Day 3 (N)	2.8 ± 3.3 (6), − 0.6 to 6.2	5.1 ± 5.1 (10), 1.5–8.7	− 2.3, − 7.3 to 2.7	0.346
Day 4 (N)	2.3 ± 4.3 (6), − 2.2 to 6.8	4.8 ± 5.7 (9), 0.4–9.1	− 2.4, − 8.3 to 3.5	0.386
Revised Atlanta classification severe	35.0% (7), 15.4–59.2%	26.8% (5), 9.1–51.2%	8.7%, − 21.2% to 38.8%	0.731
Highest prognostic score	3.6 ± 1.9, 2.7–4.5	2.8 ± 1.8, 2.0–3.7	0.8, − 0.4 to 2.0	0.210
Highest CRP (mg/dL)	27.9 ± 9.5, 23.4–32.3	24.9 ± 8.6, 20.7–29.0	3.0, − 2.9 to 8.9	0.303
Duration of SIRS positivity (days)	4.0, 2.0–5.0	2.0, 1.0–5.0	− 1.0, − 3.0 to 0.0	0.108
Necrosectomy	0% (0), 0.0–16.8%	5.3% (1), 0.1–26.0%	− 5.3%, − 35.3 to 25.7%	0.487
Survival				0.605
Day 30 (N at risk)	100.0% (20)	94.7% (18)		
Day 60 (N at risk)	95.0% (19)	94.7% (18)		
Day 90 (N at risk)	90.0% (18)	94.7% (18)		

Data are shown as percentage (*n*), 95% CI; mean ± standard deviation, 95% CI; or median 95% CI. Differences and 95% CIs between the groups are shown in the “Difference” column. Pancreatic necrosis was assessed by blinded central review of contrast-enhanced CT images obtained on Day 14 after the start of the study drug administration. CTSI is the CT severity index. CFC is the cumulative fentanyl consumption. AFA is the additional fentanyl administration. The numbers of participants who underwent pain assessment in the CRAI group were nine on Day 2, six on Day 3, and six on Day 4, while the corresponding numbers in the IV group were 11 on Day 2, 10 on Day 3, and nine on Day 4

#### Secondary endpoints

Outcomes related to the secondary endpoints are summarized in Table 2. Except for 24-h CFC and AFA, no superiority of CRAI was demonstrated. Data on 24-h CFC and AFA on Day 2, Day 3, and Day 4 under the IV-PCA pain management protocol (Suppl.) were compared between the two groups. At baseline, pain assessed by NRS in IV-PCA participants was comparable between the groups (Table 1). In the CRAI group, 24-h CFC on Day 2 and Day 3 was significantly less than in the IV group; CRAI demonstrated superiority. However, the between-group difference in 24-h CFC on Day 4 was not significant. The 24-h AFA on Day 2 in the CRAI group was significantly less than that in the IV group; CRAI demonstrated superiority. However, the 24-h AFA values on Day 3 and Day 4 were not significantly less in the CRAI group than in the IV group.

### Safety analysis

One serious adverse event occurred in the IV group, and overall there were three deaths, two in the CRAI group and one in the IV group (Suppl. Table 1). It was determined that there was no causal relationship between any of these events and the study drug or treatment methods. There were two significant adverse events related to CRAI therapy; one was bleeding from the catheter insertion site and the other was splenic infarction (Suppl. Table 2).

## Discussion

The results of this trial demonstrated that the efficacy of CRAI of NM to inhibit pancreatic necrosis development was not superior to that of IV NM. Originally, the main purpose of CRAI therapy with protease inhibitors was to inhibit the development of pancreatic necrosis through an anticoagulant effect and by blocking trypsin, which induces vascular endothelial cell damage. It was shown that development of irreversible necrosis occurred within 3–5 days after the onset of AP [5, 6, 22]. In a previous randomized, controlled study, Piaścik et al. demonstrated that if CRAI of NM was started within 72 h after the onset of AP, it significantly improved CTSI scores [14]. This indicated that early CRAI of NM could effectively inhibit the development of pancreatic and peripancreatic necrosis. According to the selection criteria in the present trial, the average time from the onset of AP to the start of the study drug administration was 37.3 h in the CRAI group and 32.9 h in the IV group. There was no significant difference between the two groups (Table 1). In this trial, all participants began receiving NM within 72 h after the onset of AP. Because the study drug (NM) was directly supplied to the pancreas by CRAI therapy and this maintained the drug concentration at a high level in the pancreatic tissue, it was expected that development of pancreatic necrosis would be more effectively inhibited by CRAI of NM than by IV NM. Unexpectedly, however, CRAI was not effective with regard to inhibiting the development of pancreatic necrosis.

Two hypotheses are suggested to explain this unanticipated finding. The first is that fluid management protocols have become well established in clinical practice over the last 15 years, and have remarkably improved the prognosis of patients with SAP. Rapid infusion of Ringer’s solution in the early phase of AP can stabilize hemodynamics and maintain pancreatic perfusion. It is possible that the IV administration of NM is comparable to CRAI in terms of maintaining the drug concentration at a therapeutic level in the pancreas and effectively inhibiting the progression from pancreatic ischemia to necrosis. The second reason is that a trypsin- and ischemia-independent mechanism of pancreatic necrosis may exist. Recent basic research demonstrated that a novel TNF-alpha-dependent mechanism of inducing acinar cell necrosis is involved in the pathogenesis of AP [23, 24]. A cerulein-induced murine AP model demonstrated that programmed necrosis regulated by PIPK1, RIPK3, and MLKL, which are intracellular molecules that play a role in TNF-alpha receptor signaling, participated in acinar cell necrosis in the early phase of AP [24, 25]. This programmed cell death, called necroptosis, induces strong inflammation around the dead cells, which is a characteristic that differs from apoptosis. A large amount of cytotoxic substances called DAMPs, including high-mobility group protein B1 (HMGB1), DNA, and heat shock protein (HSP), is released from acinar cells entering necroptosis and is involved in the development of SAP [23]. If this mechanism is a primary means of deciding the fate of acinar cells in the early phase of AP, the ability of protease inhibitors to inhibit the development of pancreatic necrosis may be limited.

The superior analgesic effect of CRAI of NM was demonstrated in this trial. In participants who received fentanyl via IV-PCA, the 24-h CFC on Day 2 and Day 3 and the 24-h AFA on Day 2 were significantly lower in the CRAI group than in the IV group. Several studies reported the analgesic effect of CRAI therapy with protease inhibitors, and demonstrated that in most cases, severe pain caused by AP disappeared within a few days after the start of CRAI therapy [10, 26, 27]. Ino et al. conducted a prospective, non-randomized, controlled study in participants receiving CRAI of 2400 mg/day of GM and non-CRAI controls, and found that the duration of pain was significantly shorter in the CRAI group than that in the control group (1.9 ± 0.26 days vs. 4.3 ± 0.5 days, respectively, *P* = 0.0005) [27]. The mechanisms of the analgesic effect of CRAI therapy with protease inhibitors are speculated to involve a direct inhibitory effect of ectopically activated trypsin, reversal of pancreatic ischemia, and inhibition of protease-activated receptor-2 (PAR-2) signaling [9, 28, 29]. Despite the superior analgesic effect of CRAI of NM in the early phase of AP in this trial, after the end of the study drug administration the percentage of participants who used fentanyl was similar in the CRAI and IV groups (55.0% vs. 31.6%, respectively). The analgesic effects of CRAI therapy seemed to be limited to the period of study drug administration; pain recurred thereafter, with the intensity depending on the degree of organ damage and inflammation.

This trial showed that CRAI of NM was superior to IV NM in terms of pain relief, although this effect was restricted to the early phase of SAP. By contrast, CRAI therapy was not superior in terms of reducing the extent of pancreatic necrosis. Moreover, the improvement of systemic inflammation and organ damage was comparable in the CRAI and IV groups. Arterial catheter-related adverse events occurred in two patients (overall incidence, 10%), namely bleeding from the injection site and splenic infarction. In summary, CRAI of NM was not superior to the less-invasive IV NM, but was associated with a higher safety risk.

This trial had several limitations. First, the sample size was relatively small. Because this study was planned as an exploratory clinical trial to obtain data necessary for a future verification clinical trial, the sample size was determined according to the number of participants who could realistically be enrolled within about 1 year. The CRAI and IV groups showed no differences in the primary endpoint or in secondary endpoints other than analgesic effect; therefore, our initial hypothesis was disproven. As a result, we considered that our results justified abandoning the verification trial. Second, the open-label design was a limitation; however, because CRAI is an invasive treatment, we could not conduct a double-blind trial for ethical reasons. To minimize bias, imaging data related to the primary endpoint were evaluated centrally by three radiologists who were blinded from the clinical data and allocation information. Objective pain assessment was performed using 24-h CFC and AFA measured by IV-PCA to minimize bias, but selection bias existed nonetheless. Finally, the selection of participants who were expected to develop pancreatic necrosis was difficult. It was described to be impossible to predict pancreatic necrosis using contrast-enhanced CT within 3 days after the onset of AP [5]. On the other hand, because CRAI therapy must begin before the development of pancreatic necrosis, the participants in this trial had to be selected within 3 days after AP onset. This posed a dilemma regarding participant selection. In this trial, we used widely accepted Japanese severity criteria for the CT imaging findings, namely those involving LEPP (Fig. 1), which can be detected within 48 h after the onset of AP and which was shown to be associated with AP morbidity and mortality [21, 30]. In the clinical setting, LEPP is frequently indicative of pancreatic ischemia. However, intestinal edema and adipose deposition into the pancreatic parenchyma are known to be additional factors that reduce CT values to below the normal range. Perfusion CT is a candidate modality for detecting ischemic pancreatic parenchyma in the early phase of AP [31]. Although perfusion CT can objectively evaluate pancreatic blood flow, there are several drawbacks so far; for example, the analysis algorithm varies among CT manufacturers, most CT devices cannot analyze the perfusion of the entire pancreas during a single examination and in many facilities perfusion CT cannot be performed in emergency situations. Therefore, perfusion CT could not be used in this multicenter trial. As there is no other realistic choice for diagnosing pancreatic ischemia, we used contrast-enhanced CT, which could be performed in all participating facilities at any time. In general, the CT value of pancreatic necrosis on contrast-enhanced CT is below 30 HU, while that of normal pancreatic parenchyma is above 100 HU [22]. In this study, LEPP was considered present if the mean CT value of a ROI was below 70 HU, which is an intermediate value between those of necrotic and normal tissue. The defined criteria were successfully used to select approximately 20% of participants who developed pancreatic necrosis involving more than one-third of the pancreas. This was possible even though these participants received infusion therapy, which is recommended by published guidelines, along with NM administration. Consequently, the selection criteria of this trial had some validity. However, the severity of AP in this trial was somewhat less than in a previous retrospective report that demonstrated the efficacy of CRAI therapy in decreasing mortality [17]. In that report, disease severity was extremely high, with mortality ranging from 14 to 20%. Although the results of this trial may have been different if participants with extremely severe SAP were selected, such critically ill patients are rare and there is no reliable, established method to diagnose severity in the very early phase of AP. It would be extremely difficult to conduct a prospective clinical trial targeting such critically ill patients with early AP.

## Conclusion

This is the first randomized, controlled trial designed to compare CRAI and IV administration of the same dose of NM, and it therefore addressed the true efficacy and safety of CRAI therapy in patients with predicted SAP. The results demonstrated that CRAI of NM was not superior to IV NM. Rather, IV NM can be an effective therapy for predicted SAP, with efficacy comparable to CRAI of NM and an excellent safety profile.

## Electronic supplementary material

Below is the link to the electronic supplementary material.

Supplementary material 1 (DOCX 202 kb)

Supplementary material 2 (DOCX 23 kb)

## Abbreviations

SAP: Severe acute pancreatitis
CRAI: Continuous regional arterial infusion
IV: Intravenous
NM: Nafamostat mesilate
DAPMs: Damage-associated molecular pattern molecules
CTSI: Computed tomography severity index
SIRS: Systemic inflammatory response syndrome
LEPP: Low enhancement of the pancreatic parenchyma
HU: Hounsfield unit
IV-PCA: Intravenous injection of fentanyl by patient-controlled analgesia
NRS: Numerical rating scale
RASS: The Richmond Agitation-Sedation Scale
CFC: Cumulative fentanyl consumption
AFA: Additional fentanyl administration
CI: Confidence interval
