# Supplementary material for: Continuous regional arterial infusion versus intravenous administration of the protease inhibitor nafamostat mesilate for predicted severe acute pancreatitis: a multicenter, randomized, open-label, phase 2 trial
Source: J Gastroenterol. 2019 Nov 22;55(3):342–52. doi: 10.1007/s00535-019-01644-z (PMC7026212; doi:10.1007/s00535-019-01644-z)
Supplement: Supplementary file 1 — Supplementary material 1 (DOCX 202 kb) [file 535_2019_1644_MOESM1_ESM.docx]

Continuous regional arterial infusion versus intravenous administration of the protease inhibitor nafamostat mesilate for predicted severe acute pancreatitis:

A multicenter, randomized, open-label, phase 2 trial

**Protocol**

**Principal investigator**

**Tohoku Medical and Pharmaceutical University**

**Morihisa Hirota**

**Tohoku University Graduate School of Medicine**

**Tooru Shimosegawa**

Protocol number: C-21

version 1.8

April 24^th^ 2017

**Background and Aim**

Continuous regional arterial infusion (CRAI) of the protease inhibitor nafamostat mesilate (NM) is used in the context of predicted severe acute pancreatitis (SAP) to prevent the development of pancreatic necrosis. Although this therapy is well known in Japan, its efficacy and safety remain unclear.

This trial is designed to demonstrate the clinical efficacy and safety of CRAI therapy with NM in patients with predicted SAP using a control group treated with continuous intravenous (IV) administration of NM at the same dose as the study group.

**Trial design**

An investigator-initiated and -driven, multicenter, open-label, randomized, controlled phase 2 superiority trial.

Study participants are randomly assigned in a 1:1 ratio either to a study group or a control group.

The primary endpoint is the percentage of participants in each group with a large area of pancreatic necrosis after the study treatment, as determined by blinded central review. The secondary endpoints are outcomes related to inflammation, pain, morbidity, and mortality.

**Trial period, Case registration period**

Trial period; February 1^st^ 2016 – March 31^st^ 2018

Case registration period; March 1^st^ 2016 – December 31^st^ 2017

**Sample size**

Study group; 20

Control; 20

**Trial drug**

- - Trial drug: FUT-200
  - Nonproprietary name: Nafamostat Mesilate
  - Chemical name: 6-Amidinonaphthalen-2-yl 4-guanidinobenzoate bis (methanesulfonate)
  - Molecular formula: C_19_H_17_N_5_O_2_・2CH_4_O_3_S
  - Molecular weight: 539.58
  - Strage method: Shading and room tmparature(1℃～30℃)

| Trial drug | Code | Content・Dosage |
| --- | --- | --- |
| FUT-200 | FUT-200-50 | Contains 50mg of nafamostat mesilate per vial・Injrction |
|  | FUT-200-10 | Contains 10mg of nafamostat mesilate per vial・Injrction |

- Tohoku University purchased the trial drug nafamostat mesilate from TORII Pharmaceutical.

**Allocation**

Randomization is performed by dynamic allocation (minimization method) with the following allocation factors:

study facility,

etiology (alcoholic),

contrast-enhanced CT grade 3.

**Selection criteria**

1. Patients are eligible for this trial if they are diagnosed with SAP according to the Japanese severity criteria: low enhancement of the pancreatic parenchyma (LEPP) in at least one of three pancreatic sections on contrast-enhanced CT (grade 2 or 3) within 48 hours after the onset of abdominal pain

LEPP is defined if the mean CT value of a maximum region of interest (ROI) in one of three pancreatic sections is less than 70 HU.

1. Age more than 20, or 79 and less.
2. Study drug administration is required to begin within 24 hours after the CT.
3. In principle, written informed consent is obtained from both the patient and an acceptable legal representative, but if the patient him- or herself had difficulty in communication, the consent is obtained from the legal representative only.

**<definition of AP>**

AP is diagnosed if at least two of the following three clinical features are present: typical abdominal pain, abnormally high levels of serum pancreatic enzymes, and characteristic findings of AP on cross-sectional abdominal imaging.

**<Definition of the time of onset>**

The time of onset of acute pancreatitis (AP) is defined as the time when severe abdominal pain or back pain occurs. If there is pre-existing mild pain, the time when the pain becomes markedly more severe is defined as the time of onset. Based on the history obtained from the patient, the principal investigator or subinvestigator will determine the time of onset as a specific hour of the day.

**<Contrast-enhanced CT>**

**Recommended CT procedure:**

Contrast-enhanced abdominal CT scans with a slice thickness of 5 mm or less are obtained by MDCT. After plain CT scans are taken, rapid intravenous infusion of a nonionic contrast agent (300–370 mgI/mL) is performed at 3.0–4.0 mL/sec (the dose is based on the patient’s body weight) and dynamic scans are obtained, including pancreatic parenchymal phase images, hepatic portal phase images, and equilibrium phase images at 40 seconds, at 70 seconds, and at 120 seconds after the start of contrast infusion, respectively. The region from the superior margin of the liver to the pelvic floor is scanned in the equilibrium phase or the other phases. In patients with an eGFR ≥ 30 mL/min/1.73m^2^ and < 45 mL/min/1.73m^2^, the contrast agent dose should be decreased to 80% of that based on body weight, and an adequate amount of fluid should be infused intravenously.

- - Together with contrast-enhanced CT scans obtained on Day 14, abdominal CT scans obtained within 48 hours after the onset of AP are submitted to the independent evaluation committee to allow review of the patient’s eligibility for this study. If multiple abdominal CT scans with sufficient quality to evaluate the contrast-enhanced CT Grade were obtained within 48 hours after the onset of AP, the scans taken at the nearest time to enrollment are submitted.

As well as patients who are ineligible for other reasons, patients with poor quality CT scans whose eligibility cannot be evaluated accurately should be excluded from this study.

**Exclusion criteria**

1. Treatment with CRAI of a protease inhibitor or anticoagulant agent before informed consent is obtained.
2. IV administration of NM at 240 mg/day or more before informed consent is obtained.
3. Inability to specify the onset of abdominal pain.
4. Suspected malignancy of the pancreas.
5. History of pancreatectomy.
6. Estimated glomerular filtration rate of less than 30 mL/min/1·73m^2^.
7. Serum potassium level of 5·5 mEq/L or higher.
8. Contrast agent allergy.
9. Pregnancy.
10. Age less than 20, or 80 and over.
11. Deemed unsuitable for study participation by an investigator due to serious comorbidity.

**Administration of the study drug**

Dosage and administration of the study drug in the arterial infusion (CRAI) group and the control group are explained below. The study drug is administered for 5 consecutive days from Day 1 to Day 5. Day 1 is defined as the 24-hour period from initiation of the first dose of the study drug.

**CRAI**

FUT-200 (240 mg; the daily dose) is dissolved in 5% glucose solution. The total drug solution volume for daily infusion should be 480 mL and the solution is delivered by continuous arterial infusion over 24 hours.

**Control (IV)**

FUT-200 (240 mg; the daily dose) is dissolved in 5% glucose solution. The total drug solution volume for daily infusion should be 960 mL and the solution is delivered by continuous intravenous infusion over 24 hours.

**Concomitant drugs: antibacterial agents and IV-PCA**

**1.** **Antibacterial agents:**

- In all patients, meropenem (0.5 g) is diluted in normal saline, etc. and is administered intravenously three times a day from Day 1 to Day 5.
- If the specified treatment with meropenem cannot be performed for a valid medical reason (e.g., allergy to meropenem) or the patient develops an adverse reaction during treatment and discontinues meropenem because a causal relationship cannot be excluded, administration of another antibacterial agent is allowed (preferably another carbapenem). In this case, the reason for discontinuing meropenem, the name of the antibacterial agent used instead of meropenem, and the date of discontinuing meropenem / starting the other antibacterial agent should be recorded.
- In patients with renal impairment, the dose and dosing interval of meropenem should be adjusted as follows:

If Ccr is 26–50 mL/min: meropenem should be administered at 0.5 g twice a day.

If Ccr is 10 to < 26 mL/min: meropenem should be administered at 0.25 g twice a day.

If Ccr is < 10 mL/min: meropenem should be administered at 0.25 g once a day.

**2.** **Pain management with IV-PCA:**

See Supplemental 4. “Protocol for pain management with IV-PCA”.

**Prohibited concomitant drugs**

Other protease inhibitors (nafamostat mesilate preparations other than the study drug, and gabexate mesilate, ulinastatin, and camostat mesilate).

**Prohibited concomitant treatments**

Patients are prohibited from receiving arterial infusion of any antibacterial agent, arterial infusion of any protease inhibitor other than the study drug, and arterial infusion of any anticoagulant throughout the study period.

**Endpoints**

- 1. **Primary endpoint**

The primary endpoint is a comparison of the percentage of participants in the CRAI and IV groups who develops a large extent of pancreatic necrosis, variably defined in two different analyses as more than one-third of the pancreas or more than two-thirds of the pancreas, as shown by contrast-enhanced CT on Day 14.

- The imputation of missing data for the primary endpoint is defined in advance for participants who can not undergo contrast-enhanced CT on Day 14 due to progressive and persistent renal failure or death.
- All the CT images are collected and analyzed by three radiologists who are blinded to the clinical and allocation information.
  1. **Secondary endpoints**

CT severity index (CTSI) estimated by CT images on Day 14

CTSI is analyzed by blinded central review

A comparison of 24-hour cumulative fentanyl consumption (CFC) and additional fentanyl administration (AFA) on Day 2, Day 3, and Day 4 in participants who received IV-PCA.

The percentage of participants diagnosed with SAP according to the modified Marshall score defined in the revised Atlanta classification between Day 1 and Day 5.

The highest prognostic score according to the Japanese severity classification between Day 1 and Day 5.

The highest level of CRP between Day 1 and Day 5.

The duration of SIRS positivity between Day 1 and Day 5.

Necrosectomy rate up to Day 90.

Mortality up to Day 90.

**Safety endpoints**

1. Adverse events
2. Clinical laboratory test results
3. Vital signs (body temperature, blood pressure, heart rate, respiratory rate, and SpO_2_ or SaO_2_)
4. Safety assessment of catheter placement for arterial infusion:
5. Puncture site bleeding that requires hemostasis or blood transfusion [yes/no].
6. Ecchymoses requiring treatment [yes/no].
7. Displacement of the catheter [yes/no].
8. Occlusion of the catheter or other abnormalities [yes/no].
9. Evaluation of circulation in the lower extremity with the arterial infusion catheter.

**Inputting adverse events into the electronic case report form**

In principle, each adverse event should be input into the eCRF by using the name of the condition/disease that is diagnosed. If a diagnosis cannot be made or if the principal investigator/subinvestigator decides that it is reasonable to not enter the name of a specific condition/disease, the adverse event should be input into the eCRF as a symptom or sign (including an abnormal laboratory test result).

The principal investigator/subinvestigator should check the medical records, etc. to ensure that all adverse events for evaluation (including serious adverse events) are recorded accurately and should report all adverse events via the eCRF.

**Study procedures**

**Before initiation of study treatment**

**About the study treatment**

**From the first visit to initiation of study treatment**

- Regardless of whether it is before or after initiation of this study, the therapeutic strategy for acute pancreatitis should be compliant with the “Clinical practice guideline for acute pancreatitis.” Thus, medical treatment is started from the patient’s first visit or the time of diagnosis.
- Contrast-enhanced CT should be performed within 48 hours after the onset, and the severity of acute pancreatitis should be determined according to the criteria for assigning a contrast-enhanced CT Grade. Then the patient’s eligibility for this study should be evaluated.
- CHDF should be initiated for patients with severe disease, in whom hemodynamics are unstable and no urine output is observed despite adequate initial fluid therapy, and for patients with the complication of abdominal compartment syndrome (ACS).
- Patients who have received intravenous administration of any of the following drugs before enrollment can still be enrolled in this study: antibacterial agents, nafamostat mesilate or gabexate mesilate for DIC or acute pancreatitis authorized under the national health insurance scheme, or ulinastatin for acute circulatory failure or acute pancreatitis authorized under the national health insurance scheme.
- As is the case for treatment before enrollment, a protease inhibitor can be used according to the above-mentioned insurance-authorized dosage / indications during the period from enrollment to the start of study treatment, and the antibacterial agent can be continued in patients receiving one before enrollment. However, the antibacterial agent should be switched to meropenem from Day 1.

**Examinations and evaluations**

**From patient selection until enrollment**

1. The patient’s baseline characteristics are checked.
2. The results of the following tests are checked: specified laboratory tests and urine tests, 24-hour urine volume (if measured), arterial blood gas analysis, pregnancy test, chest X-ray film (mobile radiography is acceptable), ECG, vital signs (blood pressure, body temperature, respiratory rate, heart rate, and SpO_2_ or SaO_2_; these should all be measured at rest), and level of consciousness (RASS).
3. Pain is assessed (this should be done at least once before enrollment).

**From enrollment until initiation of study treatment**

1. In patients who require pain management, this is started with or without IV-PCA.
2. A peripheral blood sample is collected for measurement of the study drug concentration (first measurement).

- Measurements of the peripheral blood study drug concentration:

Measurement of the study drug concentration in the peripheral blood is performed at two time points, which are before the start of study drug administration and on Day 3 (it is expected that the blood drug concentration will reach a steady state by Day 3).

1. The vital signs should be measured and recorded within 15 minutes before initiation of study drug treatment.
2. In the CRAI group, the circulation of both lower extremities should be checked.

**Treatment period**

**Treatment protocol, examinations, and tests**

**Arterial infusion therapy**

1. Angiography and catheter placement should be performed by a doctor who is skilled in catheter manipulation. The catheter for angiographic examination is inserted from the inguinal region. Using standard angiographic procedures, angiography of the superior mesenteric artery and celiac artery is performed to observe the anatomy of these vessels. The contrast agent infusion volume and rate should be adjusted according to the vessel diameter, etc.
2. Using the angiographic images and CT scans, the artery perfusing the largest part of the ischemic region of the pancreas is selected (e.g., the superior mesenteric artery, celiac artery, or splenic artery). An indwelling catheter is advanced to the selected artery and is fixed at the inguinal region.
3. Only one catheter is placed for arterial infusion in this study.
4. FUT-200 (240 mg; the daily dose) is dissolved in 5% glucose solution. The total volume of drug solution prepared for daily infusion should be 480 mL and the drug solution is administered by continuous intra-arterial infusion over 24 hours. The dates and times of starting and finishing administration should be recorded.
5. The time of starting the first arterial infusion is defined as the start of Day 1 of the study, and the date and time of starting Day 1 should be recorded.
6. In the CRAI group, the location of the catheter should be checked on an abdominal X-ray film once every day for 5 days from the day of starting the first arterial infusion to the day before removal of the catheter (Days 1, 2, 3, 4, and 5). The catheter should be checked for displacement of the tip (whether or not the catheter tip has moved from the initial placement site) and abnormalities (e.g., breakage, twisting, or kinking). The catheter puncture site should also be inspected once a day.
7. If arterial infusion therapy is discontinued (e.g., because exchange of the catheter is required due to occlusion or other problems or to allow recovery from hyperkalemia), the patient can continue to participate in this study if arterial infusion therapy can be resumed within 12 hours after discontinuation. If discontinuation for catheter exchange or discontinuation due to hyperkalemia is required again after resuming arterial infusion therapy, the patient should discontinue this study.
8. If the study drug cannot be administered due to hyperkalemia, etc. but there is no problem with the catheter and other drugs can still be administered via it, heparinized normal saline solution (10 units/mL) should be infused at a rate of 10 mL/hr to prevent thrombus formation. If drugs cannot be administered due to a problem with the catheter such as occlusion, the catheter should be removed immediately.
9. If the catheter tip becomes displaced from the initial site, the catheter should be replaced immediately. If the study drug can still be administered via the catheter after displacement of the tip, administration is continued until catheter replacement, even though drug delivery to the ischemic area may be decreased.
10. After catheter replacement due to displacement of the tip, if displacement occurs again after catheter replacement due to displacement of the tip, the patient should discontinue this study.
11. During arterial infusion therapy, circulation should be monitored in the lower extremity in which the catheter has been inserted. If findings suggestive of acute arterial thrombosis are observed, ultrasonography, contrast-enhanced CT, angiography, etc. should be performed to confirm the diagnosis. If acute arterial thrombosis occurs, appropriate treatment should be provided.
12. If the principal investigator or a subinvestigator decides that additional examinations are required, including abdominal X-ray, inspection of the catheter puncture site, and evaluation of the circulation of the lower extremity, such additional examinations should be performed.

**Intravenous infusion**

1. FUT-200 (240 mg; the daily dose) is dissolved in 5% glucose solution. The total volume of drug solution for daily infusion should be 960 mL and the drug solution is administered via a central or peripheral vein over 24 hours. Continuous intravenous infusion is performed for 5 days, with the dates and times of starting and finishing administration being recorded.
2. If intravenous infusion is discontinued for reasons such as hyperkalemia or a problem with the central venous catheter, the patient can continue to participate in this study if intravenous infusion can be resumed within 12 hours after discontinuation. If discontinuation is required again after intravenous infusion is resumed, the patient should discontinue the study.

**During the treatment period (CRAI group and control group)**

1. The concurrent therapies specified for this study should be performed.
2. **Antibacterial agent**

In all patients, meropenem should be administered concurrently with the study drug for 5 days from Day 1 to Day 5.

1. **Pain management**

Pain management is required in patients with an NRS > 3 or CPOT score > 2. In patients with a pretreatment NRS ≤ 3 or pretreatment CPOT score ≤ 2, if the NRS exceeds 3 or the CPOT score exceeds 2 during the treatment period, pain management should be initiated according to the specified procedure. If patients with an NRS ≤ 3 or CPOT score ≤ 2 have been receiving an analgesic agent from before the start of this study, pain management should be performed for these patients after initiation of this study.

Pain management is performed with or without using IV-PCA.

1. Pain management with IV-PCA (evaluated as a secondary endpoint)

As explained above.

- - - - 1. Pain management without IV-PCA (not assessed as a secondary endpoint

If it is impossible to perform assessment of pain with the NRS or if the principal investigator or subinvestigator considers that using IV-PCA is inappropriate, pain management should be performed without IV-PCA according to the “Clinical practice guideline for acute pancreatitis”. If pain management with IV-PCA has already been performed and discontinued, patients should subsequently receive pain management without IV-PCA according to the “Clinical practice guideline for acute pancreatitis”.

1. It is permitted to perform examinations, including chest X-ray and CT, if the principal investigator or subinvestigator considers that such examinations are required.
2. During the treatment period, measurement of the specified vital signs should be performed at least 3 times every 24 hours for 5 days from Day 1 to Day 5 (specifically, at least once in each of the periods from 4:00 to noon, from noon to 20:00, and from 20:00 to 4:00 on the next morning). In addition, laboratory tests should be done at least once a day (the worst value is used), as well as urine tests once a day (and 24-hour urine volume measurement), evaluation of SIRS at least once a day for 5 days from Day 1 to Day 5 (the worst value is used), and checking of concomitant drugs / therapies. Among the items for evaluation of SIRS, the body temperature, respiratory rate, and heart rate should be measured at least 3 times every 24 hours and the value obtained when the most SIRS criteria are fulfilled should be used. If laboratory tests are performed more than once every 24 hours, the worst value is used.
3. When pain management with IV-PCA is performed, the protocol for pain management using IV-PCA (Supplement 4) should be followed.
4. If patients do not receive pain management with IV-PCA, evaluation of the RASS and NRS or CPOT score should be performed 3 times every 24 hours (at approximately 8-hour intervals) for 5 days from Day 1 to Day 5. In addition to these routine evaluations, additional evaluations should be performed if required. When a patient who is able to communicate is sleeping, the regular evaluation can be omitted. However, two consecutive regular evaluations must not be missed. In each patient, no more than one regular evaluation per day can be omitted during the period from Day 1 to Day 5.
5. In patients receiving IV-PCA, the 24-hour cumulative dose of fentanyl and the number of additional fentanyl doses per 24 hours are checked on Day 2, Day 3, and Day 4.
6. The Modified Marshall score is calculated and the severity is determined by using the score according to the revised Atlanta classification during the period from Day 1 to Day 5. If the score is determined more than once every 24 hours, the worst result for that Day is used. If any of the respiratory / kidney / cardiovascular scores is ≥ 2 for 3 consecutive days, severe disease is diagnosed.
7. Arterial blood gas analysis should be performed at least once every day from Day 1 to Day 3, and the worst result is used.
8. The prognostic factor scores are checked from Day 1 to Day 3. If the scores are determined more than once every 24 hours, the worst result for that Day is used.
9. On Day 3, a peripheral blood sample should be collected for measurement of the study drug concentration (second measurement). The blood sample can be collected at any time on Day 3.
10. If a patient develops hyperkalemia (K ≥ 5.5 mEq/L), which may be an adverse effect of nafamostat mesilate, administration of the study drug by arterial or intravenous infusion can be suspended for up to 12 hours. The K level is measured before study treatment is resumed. If K is < 5.1 mEq/L, study treatment can be resumed. If K is ≥ 5.1 mEq/L despite suspension of study treatment, the patient should discontinue this study. If the K level increases to ≥ 5.5 mEq/L again after study treatment is resumed, the patient should discontinue this study.

**Post-treatment observation period**

**Treatment protocol, examinations, and tests**

**After finishing study drug administration**

1. The arterial infusion catheter is removed immediately after completing administration of the final dose of the study drug.
2. After completion of the treatment period, standard treatment is provided according to the “Clinical practice guideline for acute pancreatitis.”
3. In principle, an antibacterial agent is not administered from Day 6.
4. Pain management is continued at the discretion of the principal investigator or subinvestigator.
5. Contrast-enhanced abdominal CT scanning and collection of a blood sample are performed on Day 14 (± 3 days). Contrast-enhanced CT scans are obtained as described above. After CT is performed, the DICOM data are submitted to the central independent evaluation committee. Along with these data, DICOM data are also submitted for the contrast-enhanced CT scans obtained within 48 hours after the onset, which were used to evaluate the patient’s eligibility for this study.
6. At 90 days after the start of study drug administration, the performance of surgery for pancreatic necrosis and the survival rate are evaluated. If a patient is discharged from hospital before Day 90, whether the patient has undergone surgery before Day 90 and whether the patient is alive at Day 90 should be determined within 7 days of Day 90 by outpatient review or by telephone interview, etc.

**Stop criteria**

1. Withdrawal of consent by the participant or the legal acceptable representative.
2. Occurrence of a serious adverse event.
3. Occurrence of serious catheter-related injuries.
4. Discovery after enrollment that the participant failed to meet eligibility criteria.
5. Participant death.

**Statistical analysis**

This study is planned as a phase 2 trial for the purpose of collecting data for future verification. Therefore, the sample size is the maximum number of participants who can be enrolled during the implementation period of about 1 year. SAP with early-phase pancreatic ischemia involving more than 30% of the pancreas is relatively rare and accounts for less than 10% of AP cases. Moreover, the number of patients who can satisfy the entry criteria and provide informed consent is expected to be small. The sample size of this trial is defined 20 for each group.

The full analysis set (FAS) is the target population for evaluating the effectiveness of this trial.

The primary endpoint is the percentage of patients with a large extent of pancreatic necrosis; the 95% confidence interval (CI) is calculated for each treatment group, and differences between groups are tested by Fisher’s exact test.

The secondary endpoints are analyzed as follows.

For CTSI, 24-hour CFC and AFA, highest prognostic score, and highest CRP level, the 95% CI of summary statistics and the mean value for each assignment group are calculated; also, the differences in mean values between the two groups are tested by Student’s t test.

The percentages of severe cases determined by the modified Marshall score are determined for the two groups, along with the 95% CIs, and the differences between the groups are tested by Fisher’s exact test.

For the duration of SIRS positivity, the medians and 95% CIs are calculated, and the differences between the groups are tested by the Mann-Whitney U test.

For necrosectomy, the ratio of the number of patients in each group who undergo necrosectomy and the 95% CIs of these ratios are calculated, and the differences between the groups are tested by Fisher’s exact test.

For overall survival from the start of the study drug administration to Day 90, the survival rates are calculated for each assignment group using the Kaplan-Meier method on Day 30, Day 60, and Day 90, and comparison between the groups is performed with the log-rank test.

A two-sided P value of less than 0·05 is considered to indicate statistical significance. Computations are performed with the use of SAS software (version 8·4).

**Supplmental 1. The severity criteria for acute pancreatitis**

1. **The Japanese severity criteria**
2. Prognostic score (severe: total prognostic score≧3 points)
3. BE≦-3mEq/L or shock (systoric blood pressure≦80mmHg)
4. PaO_2_≦60mmHg (room air) or respiratory failure (artificial respiratory ventilation)
5. BUN≧40mg/dL or creatinine≧2.0mg/dL or oliguria (uritary volume≦400mL/day after hydration)
6. LDH: more than twice higher than the upper limit of normal (700 IU/L)
7. Platelet count≦1 x 10^5^/mm^3^
8. Serum total Ca≦7.5mg/dL
9. CRP≧15mg/dL
10. Positive score of systemc inflammatory response syndrome (SIRS) criteria

≧3

1. Age≧70

- SIRS criteria
  - - 1. Body temperature: >38℃ or <36℃
      2. Heart rate: >90 bmp
      3. Tachypnea: manifested by respiratory rate >20 breaths/min or PaCO_2_ <32mmHg
      4. White blood cell count: >12000/mm^3^ or <4000/mm^3^, or the presence of >10% immature neutrophils

1. Contrast-enhanced CT criteria (severe: total severity score≧2 points)

Extension of extrapancreatic inflamematory changes

Anterior pararenal extraperitoneal space: 0 point

Root of the mesocolon: 1 point

Beyond inferior renal pole: 2 points

Low enhancement of the pancreatic parenchyma (LEPP)

(Divided the pancreas into 3 area for expediency, head, body and tail)

Limited to one area or peripancreatic area: 0 point

Extend over 2 area: 1 point

More than 2 area: 2 points

1. **Revised Atlanta criteria**

The modified Marshall score

Three organ systems should be assessed to define organ failure: respiratory, cardiovascular and renal. Organ failure is defined as a score of 2 or more for one of these three organ systems using the Modified Marshall scoring system.

| Organ system | | score | | | | | |
| --- | --- | --- | --- | --- | --- | --- | --- |
|  |  | 0 | | 1 | 2 | 3 | 4 |
| Respiratory (PaO_2_/FiO_2_） | | >400 | | 301-400 | 201-300 | 101-200 | ≦101 |
| Renal | (serum creatinine, μmol/L) | ≦134 | | 134-169 | 170-310 | 311-439 | >439 |
|  | (serum creatinine, mg/dL) | <1.4 | | 1.4-1.8 | 1.9-3.6 | 3.6-4.9 | >4.9 |
| Cardiovascular (systoric blood pressure, mmHg) | | >90 | | <90, fluid responsive | <90, not fluid responsive | <90, pH<7.3 | <90, pH<7.2 |
| For non-ventilated patients, the FiO_2_ can be estimated from below: | | | | | | | |
| Supplemental oxygen (L/min） | | | FiO_2_ (%) | | | | |
| Room air | | | 21 | | | | |
| 2 | | | 25 | | | | |
| 4 | | | 30 | | | | |
| 6-8 | | | 40 | | | | |
| 9-10 | | | 50 | | | | |

1. CT severity index（CTSI） ^4)^

The CTSI is based on findings from a CT scan with intravenous contrast to assess the severity of acute pancreatitis. The sererity of CT findings have been found to correlate well with clinical indices of severity.

| Element | Findings | Points |
| --- | --- | --- |
| Grade of AP | Normal pancreas | 0 |
|  | Pancreatic enlargement | 1 |
|  | Inflammation involving pancreas and peripancreatic fat | 2 |
|  | Single fluid collection or phlegmon | 3 |
|  | Two or more fluid collections or phlegmons | 4 |
| Degree of pancreatic necrosis | No necrosis | 0 |
|  | Necrosis of one third of pancreas | 2 |
|  | Necrosis of one half of the pancreas | 4 |
|  | Necrosis of more than one half of the pancreas | 6 |

3) Banks PA, et al. *Gut* 2013; 62: 102-111.

4) Balthazar EJ, et al. *Radiology* 1994; 193: 297–306.

**Supplemental 2. CPOT（Critical-Care Pain Observation Tool）**

| Indicator | Description | | Score |
| --- | --- | --- | --- |
| Facial expression | No muscle tension observed | Relaxed, neutral | 0 |
|  | Presence of frowning, brow lowering, orbit tightening, and levator contraction | Tense | 1 |
|  | All of the above facial movements plus eyelid tightly closed | Grimacing | 2 |
| Body movements | Does not move at all (does not necessarily mean abcent of pain) | Abcence of movement | 0 |
|  | Slow, cautious movements, touching or rubbing the pain site, seeking attention through movements | Protection | 1 |
|  | Pulling tube, attempting to sit up, moving limbs/ thrashing, not following commands, striking at staff, trying to climb out of bed | Restlessness | 2 |
| Muscle tension  Evaluated by passive flexion and extention of upper extremities | No resistance to passive movements | Relaxed | 0 |
|  | Resistance to passive movements | Tense, rigid | 1 |
|  | Strong resistance to passive movements, inability to complete them | Very tense or rigid | 2 |
| Compliance with the ventilator (intubated patients)  OR  Vocalization (extubated patients) | Alarms not activated, easy ventilation | Tolerating ventilator or movement | 0 |
|  | Alarms stop spontaneously | Coughing but tolerating | 1 |
|  | Asynchrony: blocking ventilation, alarms frequently activated | Fighting ventilator | 2 |
|  | Talking in normal tone or no sound | Talking in normal tone or no sound | 0 |
|  | Sighing, moaning | Sighing, moaning | 1 |
|  | Crying out, sobbing | Cryingout, sobbing | 2 |

5) Gelinas C, Fillion L, Puntillo KA, et al. Validation of the critical-care pain observation tool in adult patients. Am J Crit Care Med. 2001; 29: 2258-63.

**Supplemental 3. Richmond Agitation-Sedation Scale（RASS）**

| Score | Term | Description | |
| --- | --- | --- | --- |
| +4 | Combative | Overtly combative, violent, immediate danger to staff |  |
| +3 | Very agitated | Pulls or removes tube(s) or catheter(s), aggressive |  |
| +2 | Agitated | Frequent nonpurposeful movement, fights ventilator |  |
| +1 | Restless | Anxious but movements not aggressively vigorous |  |
| 0 | Alert and calm |  |  |
| -1 | Drowsy | Not fully alert but has sustained awakening  (eye opening/eye contact) to voice (≥ 10 seconds) | Verbal stimulation |
| -2 | Light sedation | Briefly awakens to voice with eye contact (<10 seconds) |  |
| -3 | Moderate sedation | Movement or eye opening to voice (but no eye contact) |  |
| -4 | Deep sedation | No response to voice but movement or eye opening to physical stimulation | Physical stimulation |
| -5 | Unarousable | No response to voice or physical stimulation |  |

1. Stawicki SP: ICU Corner Sadation scales: Very useful, very underused. OPUS 12 Scientist 2007; 1: 10-12.

**Supplemental 4.** **Pain management protocol using IV-PCA (intravenous patient-controlled analgesia)**

**Inclusion criteria**

- Study participants include those with NRS > 3 and RASS between −1 and +1. Even if NRS ≤ 3, participants with RASS between −1 and +1 and those who have already received continuous administration of analgesics are included.
- The CADD^®^-Solis PIB system is used for IV-PCA in this trial.
- One CADD^®^-Solis PIB unit is lent to each facility. If necessary, the equipment can be exchanged between participating facilities, and if a CADD^®^-Solis PIB unit is already present in the facility, the one already at the facility can be used.
- Analgesic treatment with the CADD^®^-Solis PIB can be started after enrollment in the trial. NRS and RASS must be evaluated at least once within the 24 hours before study drug administration.
- Pain assessment is performed in participants who start IV-PCA by the end of Day 1.

**Protocol for pain management by CADD^®^-Solis PIB**

Initial administration

- Fentanyl is slowly injected intravenously until initial analgesia achieves NRS ≤ 3. The maximum initial dose of fentanyl administration is 100 µg in bolus form, while the rate of continuous administration is 25 µg/hr.
- Participants with NRS ≤ 3 receive continuous intravenous fentanyl injection at 25 µg/hr with no initial bolus.
- IV-PCA settings: single dose, 25 µg; lockout duration, 10 minutes; maximum fentanyl administration dose per hour, 100 µg; maximum number of PCA administrations, four.

Maintenance dose change

- If NRS > 3 at regular pain assessment, increase the maintenance dose of fentanyl by 25 µg/hr. Regular assessment of pain and state of consciousness should be performed three times a day about every 8 hours.
- If participants complain of pain, add 25 µg of fentanyl and increase the maintenance dose of fentanyl by 25 µg/hr.
- During the lockout period, first increase the maintenance dose by 25 µg/hr. After the lockout period, if necessary, add a single dose of fentanyl.
- If the number of additional doses exceeds the maximum per hour, the investigator decides if an additional dose will be given.
- After increasing the maintenance dose, maintain that dose for at least 1 hour.
- Assuming pain is being regularly assessed three times a day, if there has been no additional administration since the previous evaluation and pain is assessed as NRS < 3, decrease the maintenance dose by 25 µg/hr. If the maintenance dose is 25 µg/hr, decrease the dose to 10 µg/hr. If the maintenance dose is 10 µg/hr, end the maintenance administration.
- The maintenance dose can be increased or decreased at the discretion of the investigator (if this occurs, record the reason).

Relapse

- If pain relapses when decreasing the maintenance dose, add a single dose of fentanyl without changing the maintenance dose. If there is still a complaint of pain after additional administration, increase the maintenance dose by 25 µg/hr.
- If the pain relapses (NRS > 3) after ending the maintenance administration, first inject fentanyl slowly until sufficient analgesia is achieved. The maximum injected fentanyl dose is 100 µg. After the initial injection, start maintenance administration of fentanyl at 25 µg/hr.

Stop criteria

- Allergy to fentanyl or other side effects.
- RASS below −2 or above +2.
- Investigator can stop IV-PCA for other reasons. If this occurs, record the reason.

**Regular evaluation of NRS and RASS**

- Participants treated with IV-PCA must undergo pain evaluation by NRS and assessment of state of consciousness by RASS at 1 hour after the start of IV-PCA, and the evaluation needs to be performed regularly every 8 hours thereafter until Day 5. If the participant is sleeping, the regular evaluation can be skipped once.
- After the end of IV-PCA, regular evaluations are performed by NRS (or the Critical-Care Pain Observation Tool) and RASS three times a day until Day 5.
- In case of pain relapse, NRS and RASS must be evaluated at 1 hour after the re-administration of fentanyl, and regularly every 8 hours thereafter until Day 5.
