# Supplementary material for: Continuous regional arterial infusion versus intravenous administration of the protease inhibitor nafamostat mesilate for predicted severe acute pancreatitis: a multicenter, randomized, open-label, phase 2 trial
Source: J Gastroenterol. 2019 Nov 22;55(3):342–52. doi: 10.1007/s00535-019-01644-z (PMC7026212; doi:10.1007/s00535-019-01644-z)
Supplement: Supplementary file 2 — Supplementary material 2 (DOCX 23 kb) [file 535_2019_1644_MOESM2_ESM.docx]

Supplemental

**Pain management protocol using IV-PCA (intravenous patient-controlled analgesia)**

**Inclusion criteria**

- Study participants include those with NRS > 3 and RASS between −1 and +1. Even if NRS ≤ 3, participants with RASS between −1 and +1 and those who have already received continuous administration of analgesics are included.
- The CADD^®^-Solis PIB system is used for IV-PCA in this trial.
- One CADD^®^-Solis PIB unit is lent to each facility. If necessary, the equipment can be exchanged between participating facilities, and if a CADD^®^-Solis PIB unit is already present in the facility, the one already at the facility can be used.
- Analgesic treatment with the CADD^®^-Solis PIB can be started after enrollment in the trial. NRS and RASS must be evaluated at least once within the 24 hours before study drug administration.
- Pain assessment is performed in participants who start IV-PCA by the end of Day 1.

**Protocol for pain management by CADD^®^-Solis PIB**

Initial administration

- Fentanyl is slowly injected intravenously until initial analgesia achieves NRS ≤ 3. The maximum initial dose of fentanyl administration is 100 µg in bolus form, while the rate of continuous administration is 25 µg/hr.
- Participants with NRS ≤ 3 receive continuous intravenous fentanyl injection at 25 µg/hr with no initial bolus.
- IV-PCA settings: single dose, 25 µg; lockout duration, 10 minutes; maximum fentanyl administration dose per hour, 100 µg; maximum number of PCA administrations, four.

Maintenance dose change

- If NRS > 3 at regular pain assessment, increase the maintenance dose of fentanyl by 25 µg/hr. Regular assessment of pain and state of consciousness should be performed three times a day about every 8 hours.
- If participants complain of pain, add 25 µg of fentanyl and increase the maintenance dose of fentanyl by 25 µg/hr.
- During the lockout period, first increase the maintenance dose by 25 µg/hr. After the lockout period, if necessary, add a single dose of fentanyl.
- If the number of additional doses exceeds the maximum per hour, the investigator decides if an additional dose will be given.
- After increasing the maintenance dose, maintain that dose for at least 1 hour.
- Assuming pain is being regularly assessed three times a day, if there has been no additional administration since the previous evaluation and pain is assessed as NRS < 3, decrease the maintenance dose by 25 µg/hr. If the maintenance dose is 25 µg/hr, decrease the dose to 10 µg/hr. If the maintenance dose is 10 µg/hr, end the maintenance administration.
- The maintenance dose can be increased or decreased at the discretion of the investigator (if this occurs, record the reason).

Relapse

- If pain relapses when decreasing the maintenance dose, add a single dose of fentanyl without changing the maintenance dose. If there is still a complaint of pain after additional administration, increase the maintenance dose by 25 µg/hr.
- If the pain relapses (NRS > 3) after ending the maintenance administration, first inject fentanyl slowly until sufficient analgesia is achieved. The maximum injected fentanyl dose is 100 µg. After the initial injection, start maintenance administration of fentanyl at 25 µg/hr.

Stop criteria

- Allergy to fentanyl or other side effects.
- RASS below −2 or above +2.
- Investigator can stop IV-PCA for other reasons. If this occurs, record the reason.

**Regular evaluation of NRS and RASS**

- Participants treated with IV-PCA must undergo pain evaluation by NRS and assessment of state of consciousness by RASS at 1 hour after the start of IV-PCA, and the evaluation needs to be performed regularly every 8 hours thereafter until Day 5. If the participant is sleeping, the regular evaluation can be skipped once.
- After the end of IV-PCA, regular evaluations are performed by NRS (or the Critical-Care Pain Observation Tool) and RASS three times a day until Day 5.
- In case of pain relapse, NRS and RASS must be evaluated at 1 hour after the re-administration of fentanyl, and regularly every 8 hours thereafter until Day 5.

**Supplemental Table 1.**

Participant deaths. Two participants in the CRAI group and one participant in the IV group died between Day 1 and Day 90. Participant No. 120 could not undergo contrast-enhanced CT on Day 14 because of renal failure. None of these participants underwent necrosectomy. The relation between the study drug or CRAI and the cause of death was not confirmed in any participant.

| No. | Assignment | Sex / Age | BMI | Cause of AP | Comorbidity | Time from onset of acute pancreatitis to study drug administration (hour) | Pancreatic necrosis | Date of death |
| --- | --- | --- | --- | --- | --- | --- | --- | --- |
| 105 | CRAI | Female / 76 | 21.2 | Post ERCP | HIV infection | 52.8 | Less than 1/3 | Day 66 |
| 120 | CRAI | Male / 70 | 18.3 | Alcohol | Hypertension | 49.0 | No data | Day 34 |
| 215 | IV | Male / 53 | 23.0 | Alcohol | Idiopathic interstitial pneumonia | 13.6 | Less than 1/3 | Day 17 |

**Supplemental Table 2.**

List of serious and significant adverse events. There was no established relationship between serious adverse events and the study drug or treatment method.

| No. | Assignment | Sex / Age | Event name | Onset time | Grade | Relationship to the study drug | Outcome | Duration (day) |
| --- | --- | --- | --- | --- | --- | --- | --- | --- |
| Serious adverse event | | | | | | | | |
| 215 | IV | Male / 53 | Acute respiratory failure | Day 4 | Grade 5 | Unrelated | Death | 14 |
| Significant adverse events | | | | | | | | |
| 104 | CRAI | Male / 58 | Bleeding from right inguinal region | Day 3 | Grade 1 | Unrelated | Recovery | 2 |
| 105 | CRAI | Female / 76 | Hyperkalemia | Day 1 | Grade 3 | Definitely related | Recovery | 13 |
|  |  |  | Hyponatremia | Day 2 | Grade 1 | Possibly related | Recovery | 12 |
| 108 | CRAI | Male / 72 | Hyperkalemia | Day 8 | Grade 2 | Unrelated | Recovery | 19 |
| 109 | CRAI | Male / 43 | Splenic infarction | Day 6 | Grade 1 | Unrelated | Alleviation | 147 |
| 113 | CRAI | Male / 78 | Hyperkalemia | Day 5 | Grade 2 | Probably related | Recovery | 11 |
| 117 | CRAI | Male / 54 | Hyperkalemia | Day 1 | Grade 1 | Definitely related | Recovery | 2 |
| 202 | IV | Male / 57 | Hyponatremia | Day 2 | Grade 4 | Probably related | Recovery | 58 |
|  |  |  | Hyperkalemia | Day 5 | Grade 2 | Maybe related | Recovery | 10 |
| 220 | IV | Male / 67 | Hyponatremia | Day 5 | Grade 3 | Unrelated | Recovery | 11 |
